# Nutrient Excess and AMPK Downregulation in Incubated Skeletal Muscle and Muscle of Glucose Infused Rats

**DOI:** 10.1371/journal.pone.0127388

**Published:** 2015-05-21

**Authors:** Kimberly A. Coughlan, Thomas W. Balon, Rudy J. Valentine, Robert Petrocelli, Vera Schultz, Amanda Brandon, Gregory J. Cooney, Edward W. Kraegen, Neil B. Ruderman, Asish K. Saha

**Affiliations:** 1 Department of Medicine, Section of Endocrinology and Diabetes, Boston University Medical Center, Boston, Massachusetts, United States of America; 2 Diabetes and Obesity Program, Garvan Institute of Medical Research and School of Medical Sciences, University of NSW, Sydney, Australia; Université catholique de Louvain, BELGIUM

## Abstract

We have previously shown that incubation for 1h with excess glucose or leucine causes insulin resistance in rat extensor digitorum longus (EDL) muscle by inhibiting AMP-activated protein kinase (AMPK). To examine the events that precede and follow these changes, studies were performed in rat EDL incubated with elevated levels of glucose or leucine for 30min-2h. Incubation in high glucose (25mM) or leucine (100μM) significantly diminished AMPK activity by 50% within 30min, with further decreases occurring at 1 and 2h. The initial decrease in activity at 30min coincided with a significant increase in muscle glycogen. The subsequent decreases at 1h were accompanied by phosphorylation of αAMPK at Ser^485/491^, and at 2h by decreased SIRT1 expression and increased PP2A activity, all of which have previously been shown to diminish AMPK activity. Glucose infusion *in vivo*, which caused several fold increases in plasma glucose and insulin, produced similar changes but with different timing. Thus, the initial decrease in AMPK activity observed at 3h was associated with changes in Ser^485/491^ phosphorylation and SIRT1 expression and increased PP2A activity was a later event. These findings suggest that both *ex vivo* and *in vivo*, multiple factors contribute to fuel-induced decreases in AMPK activity in skeletal muscle and the insulin resistance that accompanies it.

## Introduction

It has long been appreciated that nutrient excess leads to insulin resistance in many tissues [1–6]. In a recent study [6], we compared the events associated with insulin resistance in rat extensor digitorum longus (EDL) muscles incubated with a high concentration of glucose (25 vs. 5.5mM) or a normal glucose concentration (5.5mM) with added leucine (100 or 200μM) for 1h. The results strongly suggested that elevated concentrations of glucose or leucine cause insulin resistance by a common mechanism. Thus, both a high concentration of glucose and glucose plus leucine diminished AMPK activity and phosphorylation at Thr^172^ and increased mTOR/p70S6K phosphorylation. Treatment with the mTOR inhibitor rapamycin did not affect AMPK phosphorylation; however, incubation with two distinct AMPK activators, AICAR and alpha lipoic acid, prevented both the insulin resistance (impaired insulin-stimulated Akt phosphorylation) and mTOR/p70S6K phosphorylation. These results suggest that the decrease in AMPK activity is responsible, at least in part, for the glucose- and leucine-induced increases in mTOR activation and insulin resistance. In contrast, the factors responsible for the decrease in AMPK activity and whether AMPK is inhibited further with more prolonged exposure to these nutrients have not been determined.

To examine these questions, we evaluated whether several factors that have been shown to decrease AMPK activity in other settings are altered in the aforementioned EDL model in response to excess glucose or leucine for 30min-2h. Additionally, we assessed whether the same factors were changed *in vivo* in rats in which AMPK activity was diminished by a 3–8h glucose infusion that produced hyperglycemia, hyperinsulinemia, and insulin resistance. One factor examined was phosphorylation of Ser^485/491^ on AMPK’s α-subunit, an event that has been linked to the acute inhibition of AMPK by insulin within minutes in various tissues [7–9] and to the inhibition of hypothalamic AMPK by leptin [10]. Another was the upregulation of protein phosphatase 2A (PP2A), which has been shown to mediate the deactivation of AMPK in rodent aorta following the infusion of palmitate [11]. We also measured muscle glycogen content, since glycogen has been shown to inhibit AMPK in cell-free conditions by binding to the glycogen-binding domain (GBD) of its β-subunit [12]. Finally, we related diminished AMPK activity in muscle to decreases in the activity of SIRT1 and factors that regulate it. As shown by a number of groups [13–16], the activation and downregulation of SIRT1, a histone-protein deacetylase, typically parallels that of AMPK.

Intriguingly, the results revealed that all of these putative regulatory factors were altered by hyperglycemia or leucine in the incubated EDL and in muscle of the glucose-infused rats. However, the timing of the changes varied with the model, such that the initial decrease in AMPK activity generally preceded the changes in its putative regulators in the incubated muscle but not in muscle of the glucose-infused rat. Increased glycogen content was the only change temporally associated with the initial decrease in AMPK activity in the muscles incubated with high glucose or leucine, suggesting that increased cellular energy in the form of glycogen may be the initiating factor leading to AMPK inhibition by excess nutrients.

## Methods

### Ethics Statement

For muscle incubation studies performed at Boston University, protocols for animal use were reviewed and approved by the Institutional Animal Care and Use Committee of Boston University Medical Center and were in accordance with National Institutes of Health guidelines. For glucose infusion studies performed at the Garvan Institute, all surgical and experimental procedures performed were approved by the Garvan Institute/St. Vincent’s Hospital Animal Ethics Committee and were in accordance with the National Health and Medical Research Council of Australia’s guidelines on animal experimentation.

### Chemicals and materials

Antibodies for P-AMPK (Thr^172^/Ser^485/491^), P-Akt (Ser^473)^, P-GSK3β (Ser^9^), total AMPK, ACC and CAMKKβ were obtained from Cell Signaling (Danvers, MA) and P–ACC (Ser^79^) from EMD Millipore (Billerica, MA). Rabbit polyclonal anti-SIRT1 (H-300) was from Santa Cruz Biotechnology (Santa Cruz, CA). “SAMS” peptide and the polyclonal antibody used for immunoprecipitation of AMPK’s α2 catalytic subunit were obtained from QCB biotechnology (Hopkinton, MA). [γ-^32^P] ATP was obtained from Perkin-Elmer (Boston, MA) and Protein A/G plus conjugate from Santa Cruz Biotechnology (Santa Cruz, CA). All other chemicals were purchased from either Sigma-Aldrich or Fisher Scientific.

### Experimental animals

Male Sprague-Dawley rats weighing 55–65 g were purchased from Charles River Breeding Laboratories (Wilmington, MA). They were maintained on a 12:12-h light-dark cycle in a temperature-controlled (19–21°C) room and were fed Teklad Global 18% Protein Rodent Diet (Harlan, Madison, WI) and water *ad libitum*. Muscles were removed from rats anesthetized with pentobarbital (6mg/100g BW). For glucose infusion studies, adult male Wistar rats (Animal Resources Centre, Perth, Australia) were communally housed in temperature controlled (22 ± 0.5°C) room on a 12:12-h light–dark cycle. Rats were fed *ad libitum* a standard chow diet (Rat Maintenance Diet; Gordon Specialty Feeds, Sydney, Australia). After a 1 week acclimatization period, cannulae were inserted into both jugular veins.

### Muscle incubation

After removal from the rat, extensor digitorum longus (EDL) muscles were first equilibrated for 20min at 37°C in oxygenated Krebs-Henseleit solution (95% O_2_/5% CO_2_) containing 5.5mM glucose [5, 6]. They were then incubated in media containing 5.5 or 25mM glucose or with or without 100μM leucine (physiological concentration of leucine is 70–120μM) for varying time periods (30–120min) [6]. Following incubation, muscles were blotted, quick-frozen in liquid nitrogen and stored at -80°C until used for analyses. Control incubations (5.5mM glucose) were carried out for each timepoint. No temporal changes were observed in any parameters measured under this condition. For this reason, only controls at the 30 minute timepoint are shown.

### Western blot analyses

Protein homogenates (50μg) were run on a 4–15% gradient SDS polyacrylamide gel (Bio-Rad, Hercules, CA) and transferred onto a PVDF (polyvinylidene difluoride) membrane (Bio-Rad, Hercules, CA). Membranes were stained with Ponceau S (1% in 5% acetic acid) to ensure even transfer and blocked in Tris-buffered saline (pH 7.5) containing 0.05% Tween-20 (TBST) and 5% non-fat dry milk for 1h at room temperature. Next they were incubated overnight in primary antibodies (P-AMPK Thr^172^, P-AMPK Ser^485/491^, P-ACC Ser^79^, total AMPK, total ACC and SIRT1) at a 1:1,000 dilution. They were then incubated with a secondary antibody conjugated to horseradish peroxidase (GE Healthcare Bio-Sciences, Pittsburgh, PA) at a 1:5,000 dilution and subjected to an enhanced chemiluminescence solution (Pierce). Densitometry was performed using Scion Image software.

### AMPK activity assays

AMPK activity was measured in EDL muscle using the SAMS peptide assay as described previously [6, 17]. In brief, frozen muscle was homogenized and muscle lysate containing 200μg of protein was immunoprecipitated with specific antibody to AMPK’s α1 or α2 catalytic subunits and protein A/G agarose beads (Santa Cruz Biotechnology, Santa Cruz, CA). Beads were washed five times, and the immobilized enzyme was assayed based on the phosphorylation of SAMS peptide (0.2mM) by 0.2mM ATP (containing 2μCi [γ-32P] ATP) in the presence and absence of 0.2mM AMP. Label incorporation into the SAMS peptide was measured on a Racbeta 1214 scintillation counter. In addition to the SAMS peptide kinase assay, phosphorylation of αAMPK at Thr^172^ and its downstream substrate ACC Ser^79^ (which is only phosphorylated by AMPK) were used as readouts of AMPK activity.

### NAMPT activity assay

Enzymatic activity of NAMPT was assayed as described by Fulco et al (11). Muscle proteins (200μg) were suspended in 100μl 0.01mol/l NaHPO4 buffer, pH 7.4. The supernatant was removed by centrifugation at 23,000*g* for 90min at 0°C. Ten μL of supernatant were added to 50μl reaction mix (5mM Tris–HCl pH 7.4; 2mM ATP; 5mM MgCl2; 0.5mM PRPP; 6.2μM ^14^C-nicotinamide; American Radiolabelled Chemicals, St. Louis, MO) and incubated at 37°C for 1h. The reaction was terminated by transfer into tubes containing 2ml of acetone. The whole mixture was then pipetted onto acetone-pre-soaked glass microfiber filters (GF/A Ø 24mm; Whatman, Maidstone, UK). After rinsing twice with 1ml acetone, filters were dried, transferred into vials with 6ml scintillation cocktail (Betaplate Scint, PerkinElmer, Waltham, MA) and radioactivity of ^14^C-NMN was quantified in a liquid scintillation counter (Wallac 1409 DSA, PerkinElmer).

### Measurement of PP2A

The activity of PP2A was measured with the PP2A Immunoprecipitation Phosphatase Assay Kit (Millipore, Temecula, CA). Threonine phosphopeptide (K-R-Pt-I-R-R) was used as the PP2A substrate. In brief, muscles were homogenized in lysis buffer (0.5M Tris HCl, pH 7.4, 1.5M NaCl, 2.5% deoxycholic acid, 10% NP-40, 10mM EDTA) containing 1mM PMSF, and protease inhibitors. Supernatants were incubated with anti-PP2A (C subunit, clone 1D6) and protein A agarose at 4°C for 2h with constant shaking. Immunoprecipitates were then washed three times with Tris-buffered saline and diluted phosphopeptide (final concentration 750μM), and Ser/Thr assay buffer were added. The mixtures were incubated for 10min at 30°C in a shaking incubator, then briefly centrifuged and 50μl of the samples were transferred to 96-well microtiter plate. PP2A activities were determined by the addition of the Malachite Green Phosphate Detection Solution into the mixtures and measuring the absorbance at 650nm.

### Glucose Infusion

Glucose infusion was carried out as described previously [18]. Briefly, seven days after cannulation surgery, rats were randomly divided into treatment groups. After a basal blood sample (600μl) was taken, a 50% (w/v) glucose solution was infused for 0, 3, 5 or 8h using a peristaltic roller pump (101U/R; Watson-Marlow, Falmouth, UK). Blood samples were taken every 30min and the glucose infusion rate was altered to maintain a blood glucose concentration of 11mM (~16–17mM plasma glucose). Following infusion, red gastrocnemius was collected, frozen, and stored at -80°C until homogenization.

### Other analyses

Protein concentrations were determined with the bicinchoninic acid (BCA) reagent (Pierce, Rockford, IL) using bovine serum albumin as the standard. ATP, AMP, ADP, and phosphocreatine were measured spectrophotometrically as described previously [5, 6]. Lactate and pyruvate were determined spectrophotometrically using lactate dehydrogenase and NAD [6]. NAD and NADH were measured as described by Fulco et al [19]. Muscle glycogen content was determined using the phenol-sulfuric acid reaction [20].

### Statistics

Data are expressed as mean ± SEM. Statistical differences between two groups were determined by the Student’s t-test whereas multiple groups were compared by one-way analysis of variance (ANOVA) followed by Student-Newman-Keuls post hoc analysis. Differences between groups were considered statistically significant at P< 0.05.

## Results

### Studies in incubated EDL muscle: High Glucose

#### AMPK and ACC phosphorylation

**Fig 1:**
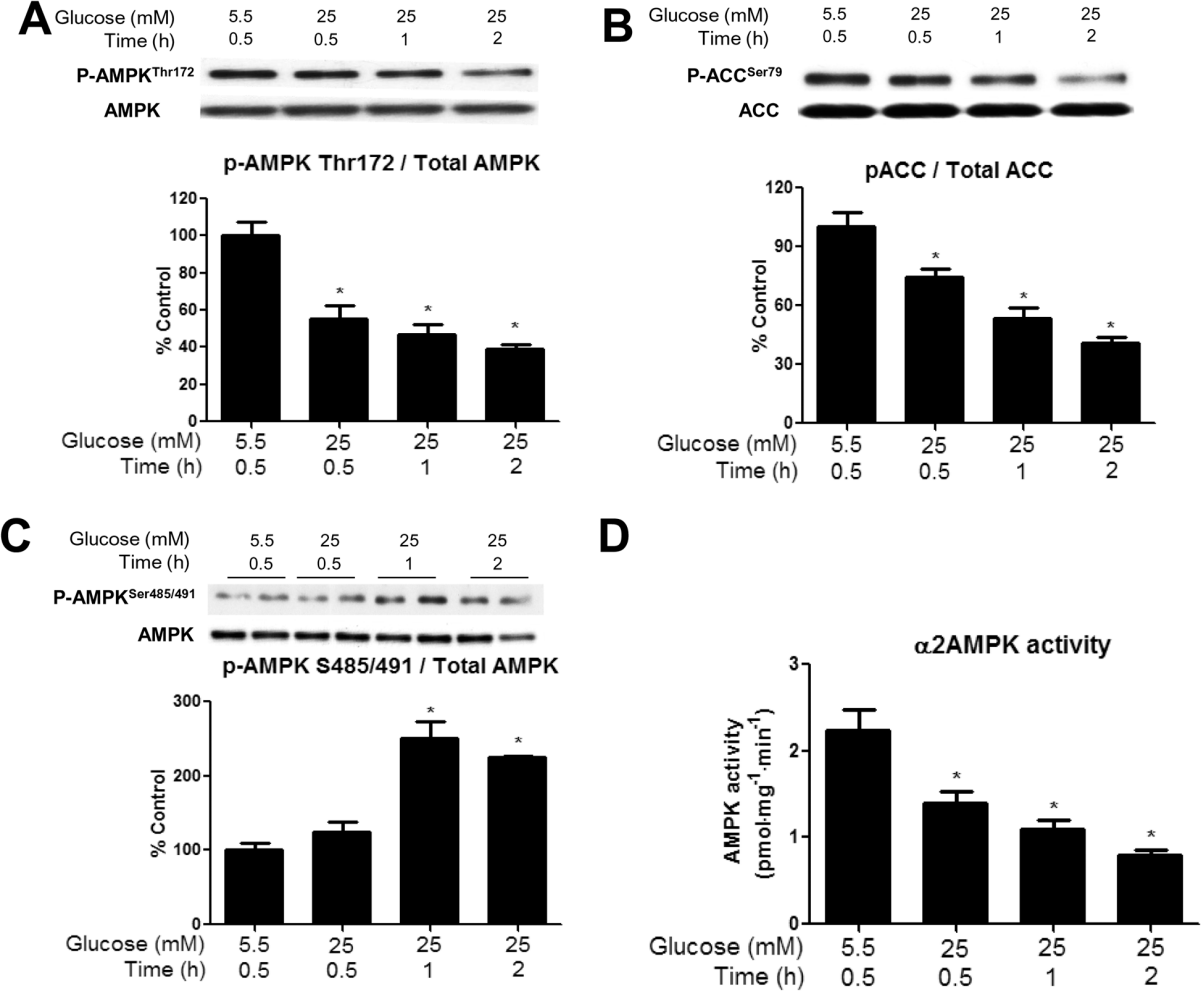
Incubation of EDL with 25mM glucose diminishes AMPK Thr^172^ phosphorylation, ACC Ser^79^ phosphorylation, and α2 AMPK activity, and increases AMPK Ser^485/491^ phosphorylation. EDL muscles were incubated in Krebs-Henseleit solution containing 25 mM glucose for 30, 60 and 120 min. Phosphorylation of AMPK Thr^**172**^ (A), ACC Ser^**79**^ (B), AMPK Ser^**485/491**^ (C) were measured by western blot and AMPK activity (D) was determined using the SAMS peptide assay. Results are means + SE (n = 6). *P < 0.05 relative to 30 min incubation with 5.5 mM glucose.

Time-course studies revealed that incubation of the EDL with 25 vs. 5.5mM glucose decreased the phosphorylation of AMPK at Thr^172^ by 40% at 30min, 50% at 60min, and 60% after 2h (Fig 1A). An almost identical pattern was observed when the activity of α2 AMPK, which is the dominant isoform in skeletal muscle was measured using the SAMS peptide assay (Fig 1D). In contrast, activity of α1 AMPK was unchanged (data not shown), indicating that the effect was isoform specific. Finally, the decrease in P-ACC Ser^79^, which is only phosphorylated by AMPK (Fig 1B), paralleled that of α2 AMPK activity. The subsequent studies examine changes in factors that may mediate this downregulation of AMPK.

#### Phosphorylation of AMPK at Ser^485/491^

Incubation of the EDL with a high glucose (25 vs. 5.5mM glucose) medium increased the inhibitory phosphorylation on AMPK at Ser^485/491^ by 250% and 200% at 1 and 2h, respectively (Fig 1C). As already noted, no insulin was added to the medium. Since Akt has previously been shown to phosphorylate AMPK at this site, we measured Akt phosphorylation. There were no significant changes in p-Akt Ser^473^ at any timepoint, but there was a trend toward increased Akt phosphorylation at 1h (p = 0.12) (S1A Fig).

#### SIRT1 expression, NAMPT activity and redox change in the presence of high glucose

**Fig 2:**
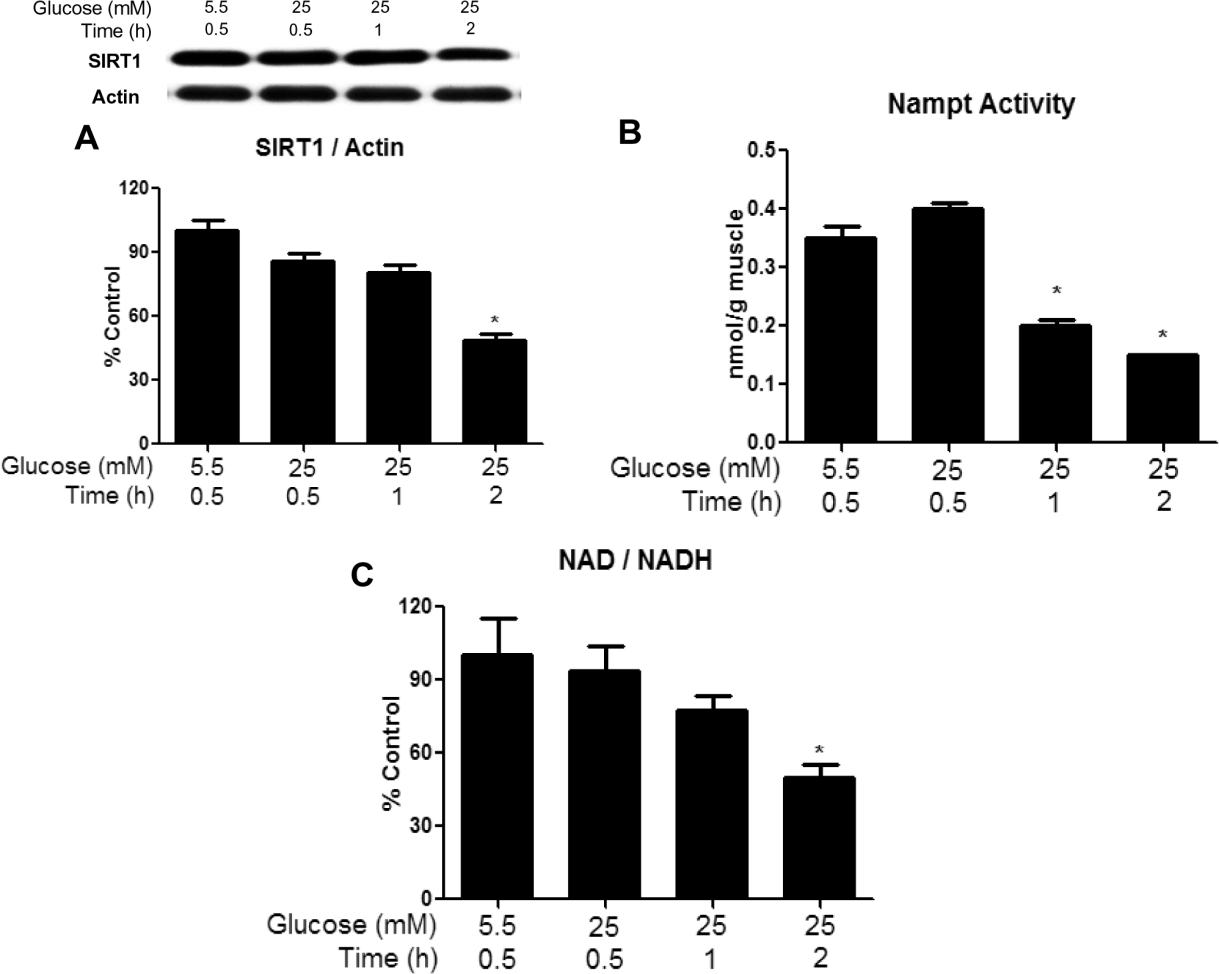
Incubation of muscle in 25mM diminishes SIRT1 protein abundance, NAMPT activity, and NAD/NADH ratio after 1 or 2h. EDL were incubated with 25 mM glucose for 30, 60 or 120 min. Western blot analysis and quantification of representative blots are shown. SIRT1 protein expression (A) NAMPT activity (B) and NAD/NADH (C) were determined as described in the methods section. Results are means + SE (n = 6). *P < 0.05 compared to 30 min incubation with 5.5 mM glucose.

SIRT1 protein expression (Fig 2A) and the NAD/NADH ratio (Fig 2C) were decreased by more than 2-fold at 2h when the concentration of glucose was increased from 5.5 to 25mM. Both tended to decrease at earlier time points, but the differences were not statistically significant. Somewhat in contrast, the activity of NAMPT (Fig 2B), a SIRT1 activator, was 50% lower in muscle incubated with 25mM glucose both at 1 and 2h, and the concentration of lactate and the lactate/pyruvate ratio were increased at both 1 and 2h (Table 1).

**Table 1:** Effects of high glucose on lactate and pyruvate.

	Lactate(μmol/mg)	Pyruvate(μmol/mg)	Lac/pyruv(μmol/mg)
5.5 mMGlucose(1 h)	13±3	1.3±0.1	10.0±2.0
25 mMGlucose(30 min)	17±2	1.6±0.1	10.6±3.0
25 mMGlucose(1 h)	31±4*	2.0±0.3*	15.5±2.0
25 mMGlucose(2 h)	33±5	2.1±0.4	15.8±2.0

Data are means ± SEM (n+4–5/group). Lactate and pyruvate are expressed as μmol/mg muscle.

*P < 0.05 relative to 30 min incubation with 5.5mM glucose

#### Cellular energy state, CAMKKβ, glycogen content, and GSK3β phosphorylation

**Fig 3:**
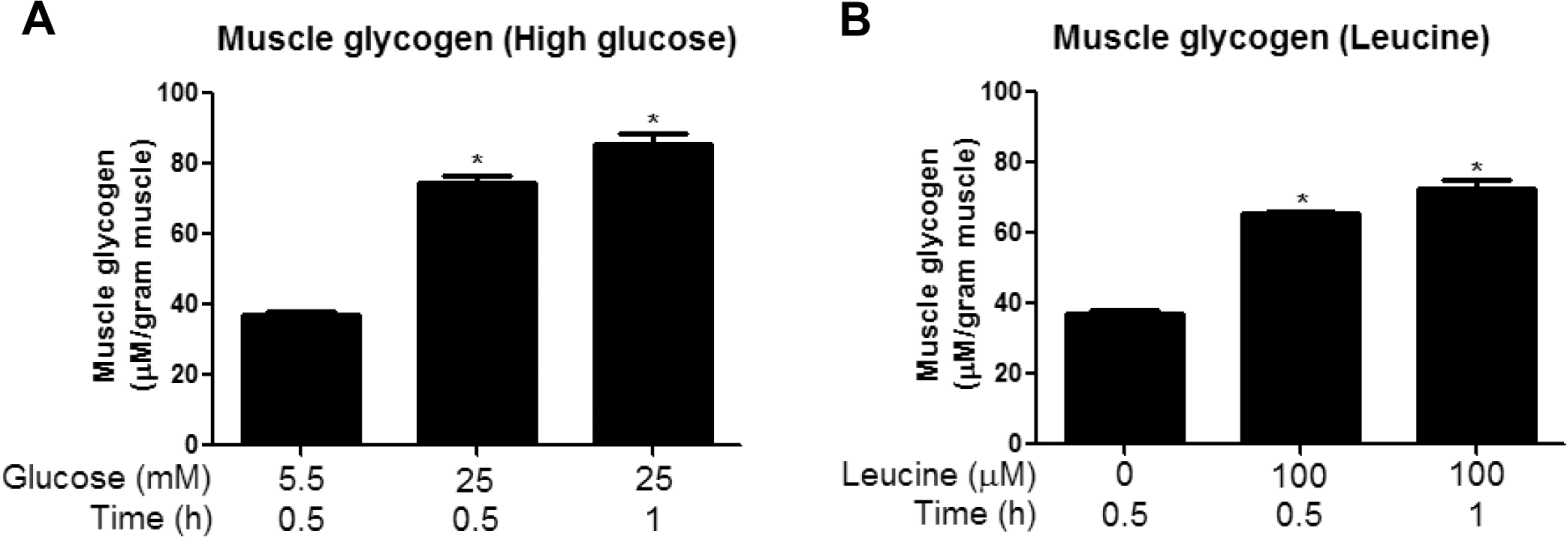
Incubation with elevated glucose levels or leucine increases muscle glycogen content at 30 min and 1h. Glycogen content was measured in EDL muscles incubated for 30 or 60 min in media containing 5.5 or 25 mM glucose (A) or 5.5mM glucose with or without 100μM leucine (B). Since no changes were found between 5.5mM glucose at 30 and 60min, only 30min values are shown. Results are means + SE (n = 4–6). *P <0.05 compared to incubation with 5.5 mM glucose.

In search for additional factors responsible for the decrease in AMPK phosphorylation caused by a high glucose concentration, we assessed cellular energy state. In keeping with previous observations [15], we found no differences in tissue concentrations of ATP, ADP, AMP or CrP in muscles incubated with 25 vs. 5.5mM glucose for 30min, 1h, or 2h (Table 2). The abundance of CAMKKβ, a known upstream kinase of AMPK, was unchanged at all timepoints measured (data not shown). Activation of another known regulator, GSK3β, which has been shown to inhibit catabolic activity of AMPK by associating with the β-subunit and phosphorylating it at Thr^479^ of the α-subunit [21], was also unchanged (S1B Fig) at all timepoints measured. Since glycogen has been shown to inhibit AMPK by associating with the CBD of the β-subunit [12], we measured muscle glycogen content following a 30 or 60 min incubation in 25 vs. 5.5mM glucose. We found that muscle glycogen was significantly increased at both timepoints, suggesting that glycogen may be responsible for the early and sustained inhibition of AMPK (Fig 3A).

**Table 2:** Effects of high glucose on adenine nucleotides.

	ATP(nmol/mg)	AMP(nmol/mg)	ADP(nmol/mg)	CP(nmol/mg)
5.5 mMGlucose(1 h)	3.9±0.04	0.06±0.003	0.60±0.04	15.0±4.0
25 mMGlucose(30 min)	3.7±0.02	0.05±0.001	0.50±0.02	13.0±2.0
25 mMGlucose(1 h)	3.9±0.06	0.04±0.001	0.55±0.04	14.0±1.7
25 mMGlucose(2 h)	3.8±0.05	0.04±0.002	0.60±0.05	13.5±2.0

Data are means ± SEM (n = 4–5/group). Nucleotide values are expressed as nmol/mg muscle.

#### Activation of PP2A in presence of high glucose

**Fig 4:**
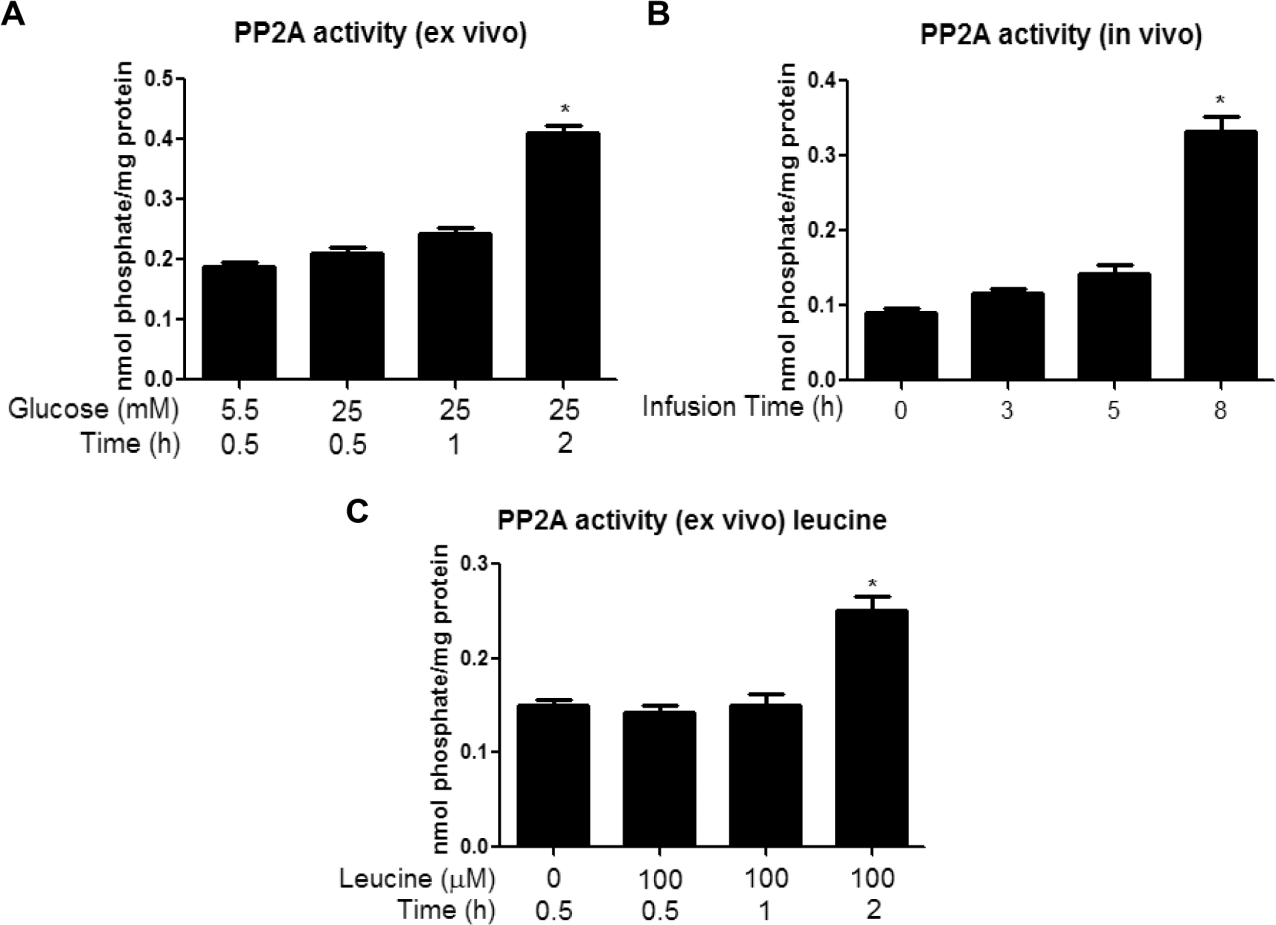
Incubation with elevated glucose levels or leucine or and *in vivo* glucose infusion increases PP2A activity at 2h (*ex vivo*) or 8h (*in vivo*). EDL muscles incubated for 30, 60 and 120 min in media containing 5.5 or 25 mM glucose (A) or 5.5mM glucose with or without 100μM leucine (C) and red gastrocnemius muscles from rats infused with glucose for 0, 3, 5, or 8h (B) were analyzed for PP2A activity as described in the methods section. Results are means + SE (n = 4–6). *P <0.05 compared to 30 min incubation with 5.5 mM glucose (A) and (C) or the 0h group for infusions (B).

PP2A is a major protein serine/threonine phosphatase that regulates AMPK, among other molecules in mammalian cells [22–24]. As shown in Fig 4A, incubation with 25 vs. 5.5mM glucose increased PP2A activity by 1.7-fold; however, as with SIRT1, this change was only observed at the 2h time point.

### Rapamycin study

**Fig 5:**
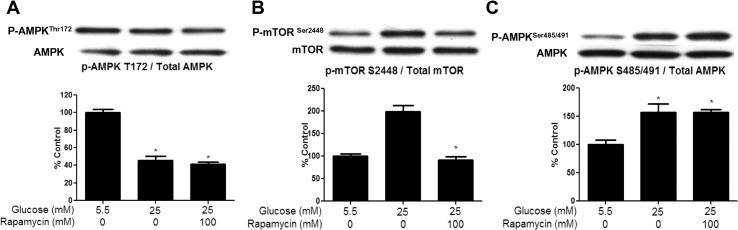
Inhibition of glucose-induced mTOR/p70S6K phosphorylation does not affect AMPK phosphorylation. EDL were preincubated in the presence of rapamycin (100μM) for 30 min and then with 5.5 or 25 mM glucose for 1hr. Muscle lysates were analyzed for P-AMPK Thr^**172**^ (A), P-mTOR Ser^**2448**^ (B) and AMPK Ser^**485/491**^ (C) by western blot. Results show quantification of western blots by densitometry. Results are means ± SE (n = 5). *, p<0.05 compared to values for 5.5 mM glucose.

We next assessed whether the high-glucose induced inhibition of AMPK was dependent on the activation of mTOR signaling. In our previous study, we found that the mTORC1 inhibitor rapamycin had no effect on a leucine-induced decrease in AMPK Thr^172^ phosphorylation. Here, we found that rapamycin (100μM) also did not prevent the reduction in P-AMPK Thr^172^ caused by incubation with 25mM glucose for 1h (Fig 5A), although the increase in mTOR Ser^2448^ phosphorylation was prevented (Fig 5B). Since mTOR/p70S6K signaling was shown to stimulate phosphorylation of AMPK at Ser^485/491^ in the hypothalamus in response to leptin, we evaluated whether rapamycin could prevent the high-glucose induced phosphorylation of this site. As shown in Fig 5C, rapamycin did not affect the increase in AMPK Ser^485/491^ phosphorylation, suggesting that mTOR/p70S6K is not responsible for the inhibitory phosphorylation in this setting.

### Studies in incubated EDL muscle: Leucine

#### Effects of leucine on AMPK Thr^172^ and AMPK Ser^485/491^ phosphorylation and other parameters

**Fig 6:**
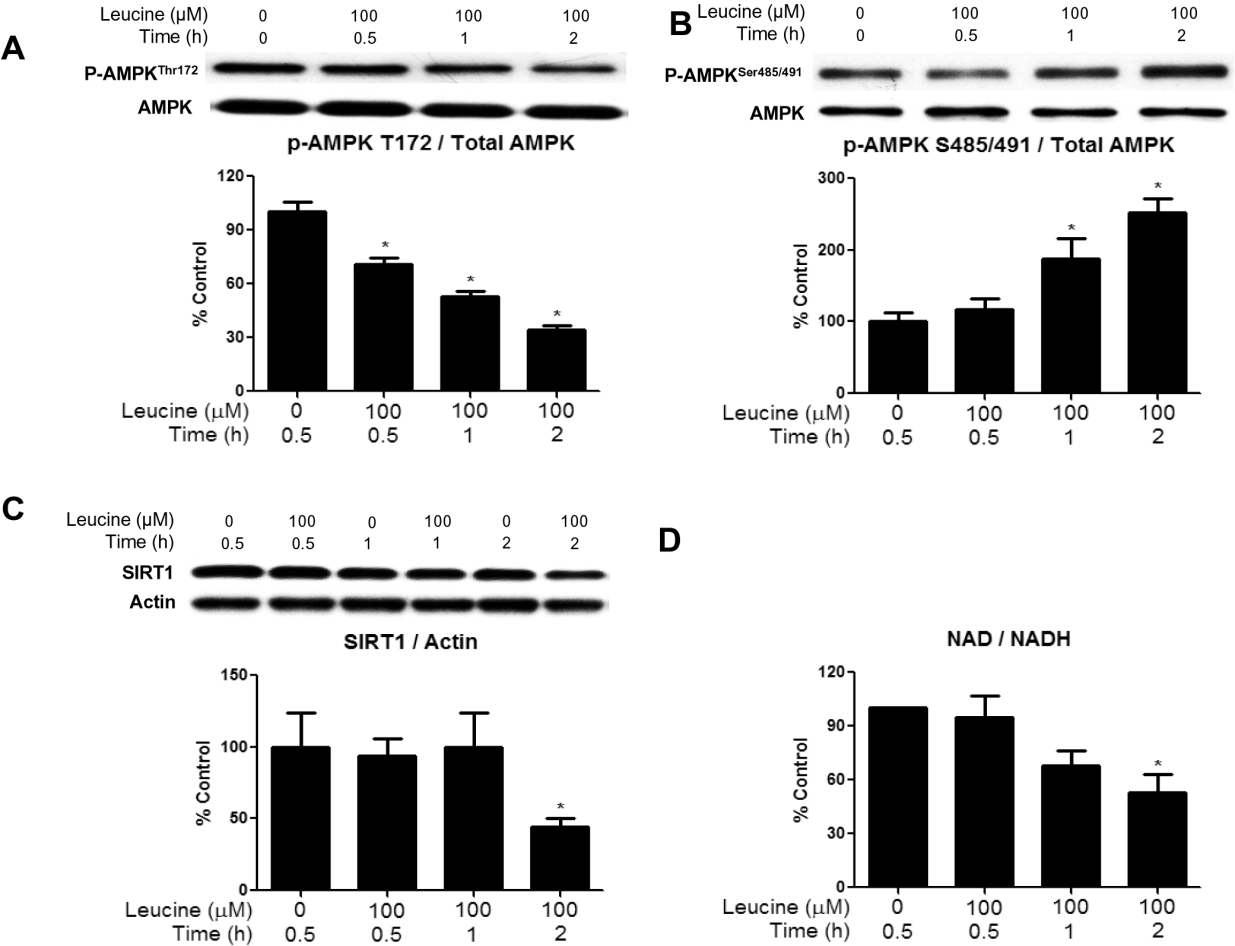
Incubation of EDL with 100 μM leucine diminishes AMPK Thr^172^ phosphorylation, SIRT1 abundance, and NAD/NADH ratio, and increases AMPK Ser^485/491^ phosphorylation. EDL muscles were incubated in Krebs-Henseleit solution containing 5.5 mM of glucose with or without 100 μM of leucine for 30, 60 and 120 min. Phosphorylation of AMPK Thr^**172**^ (A) and AMPK Ser^**485/491**^ (B), SIRT1 protein expression (C) and the NAD/NADH ratio (D) were measured. Results are means + SE (n = 6). *P < 0.05 relative to 30 min incubation with 5.5 mM glucose (without leucine).

Changes similar to those induced by 25mM glucose were observed when the EDL was incubated with 5.5mM glucose + leucine (100μM) vs 5.5mM glucose alone. They included a significant decrease in the phosphorylation of AMPK Thr^172^ at 30min with further decreases over the next 1.5h (Fig 6A). Also, AMPK Ser^485/491^ phosphorylation was increased at 1 and 2h (Fig 6B) and SIRT1 protein abundance (Fig 6C) and the NAD/NADH ratio were both decreased at 2h (Fig 6D). We also found that the addition of 100μM leucine to the medium significantly increased muscle glycogen content at 30 min and 1h (Fig 3B). Thus, the addition of a physiological concentration of leucine to a normoglycemic medium produced changes almost identical to those caused by 25mM glucose.

### Effects of a glucose infusion in vivo on rat skeletal muscle

**Fig 7:**
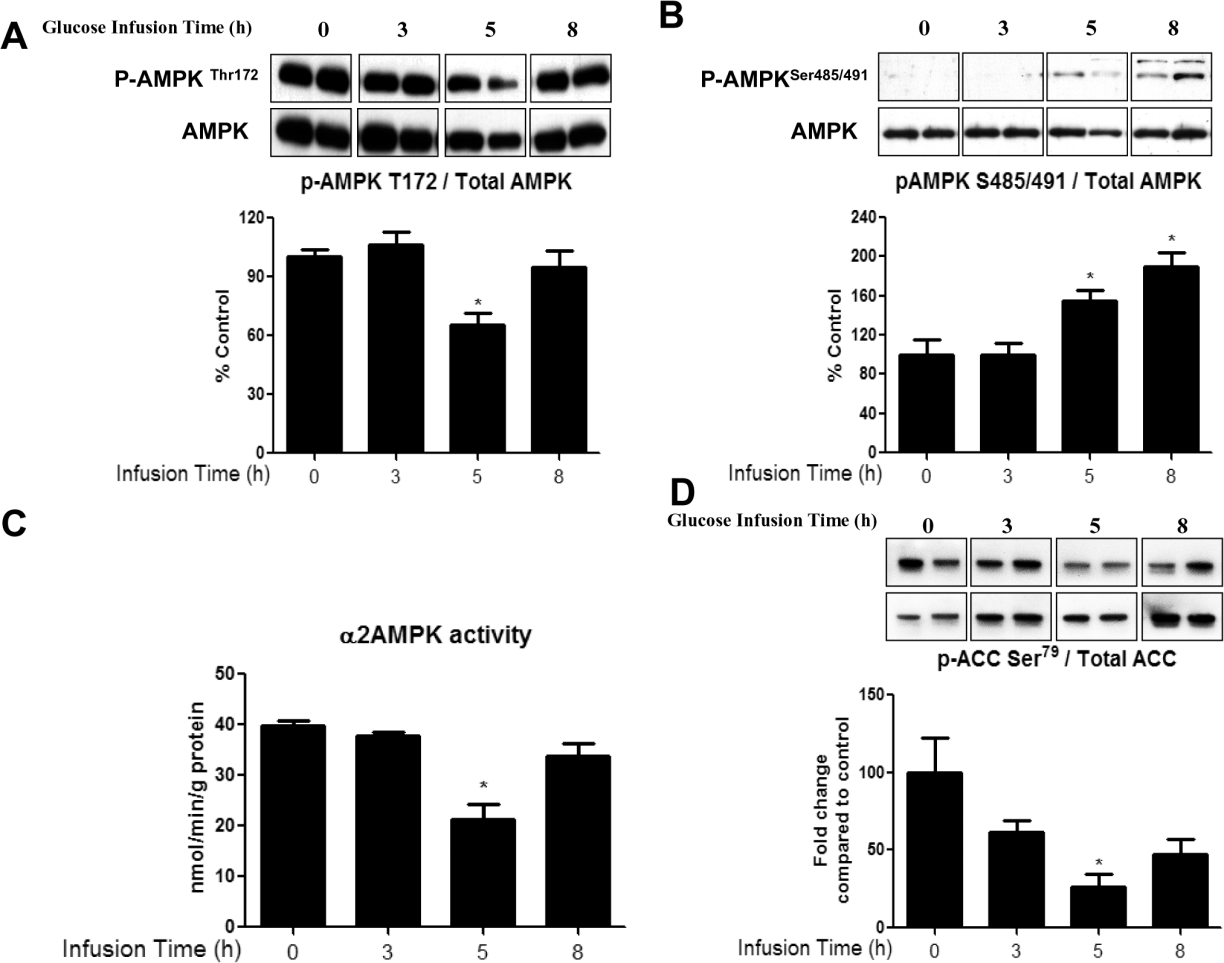
Glucose infusion decreases AMPK Thr^172^ phosphorylation and α2 AMPK activity, and increases AMPK Ser^485/491^ phosphorylation in red gastrocnemius muscle. AMPK Thr^**172**^ (A) and AMPK Ser^**485/491**^ (B) and ACC Ser^**79**^ (D) phosphorylation were analyzed by western blot. α2 AMPK activity (C) was measured using the SAMS peptide assay as described in the methods section. Data are means ± SEM. n = 4–6 rats per group, P < 0.05 vs. 0h group.

**Fig 8:**
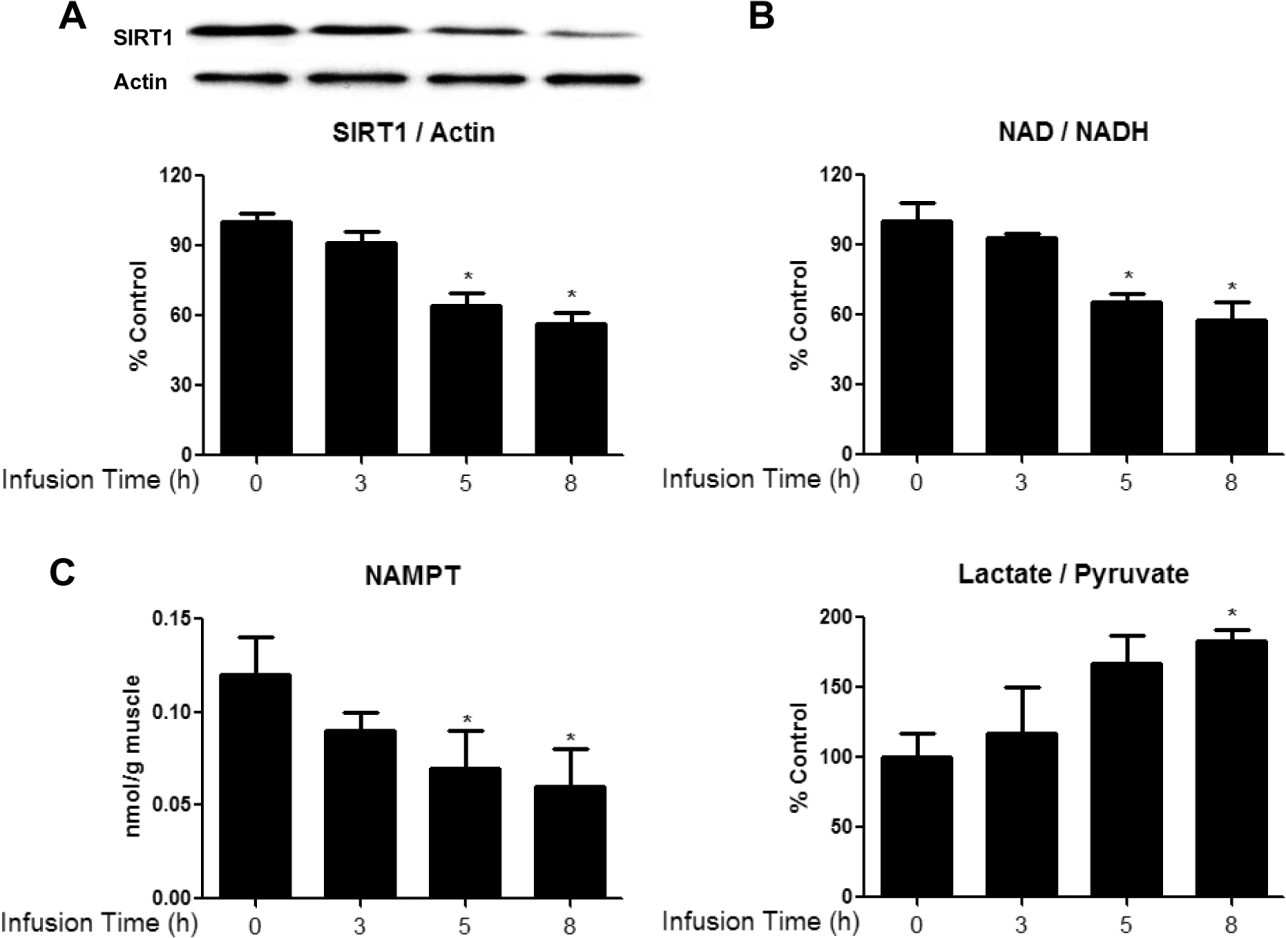
Glucose infusion diminishes SIRT1 abundance (A), NAD/NADH ratio (B), and NAMPT activity (C), and increases lactate/pyruvate ratio (D) in RG muscles. Red gastrocnemius muscles from rats infused with glucose for 0, 3, 5, or 8h were analyzed for SIRT1 protein expression (A) NAD/NADH ratio (B) NAMPT activity (C) and lactate/pyruvate (D). Data are means ± SEM. N = 4–6 rats per group, *P < 0.05 vs. 0h group.

To determine whether the effects of glucose observed in the incubated EDL also occurred in vivo and in the presence of insulin, rats were infused with 50% glucose for 0, 3, 5 or 8h at a rate adjusted to maintain plasma glucose concentration at 16–17mM. The plasma insulin level was 250μU/L during the infusion vs. 50μU/L prior to its start. In the red gastrocnemius (RG) muscle, the glucose infusion decreased both AMPK Thr^172^ phosphorylation (Fig 7A) and AMPKα2 activity (by the SAMS peptide assay, Fig 7C) with a significant change observed at 5h. Interestingly, this was followed by a secondary increase in AMPK activity, something we have not observed in previous studies [25, 26]. In keeping with these findings, the phosphorylation of ACC also was only significantly decreased at 5h (Fig 7D), although it is somewhat diminished at 3 and 8h of infusion. In contrast, the increase in phosphorylation of AMPK at Ser^485/491^ (Fig 7B) and the decreases in SIRT1 protein (Fig 8A), NAD/NADH (Fig 8B), NAMPT activity (Fig 8C) and lactate/pyruvate ratio (Fig 8D), which occurred after the decrease in AMPK-Thr^172^ in the incubated EDL, took place at the same time (5h) and were maintained at 8h. Only the increase in PP2A activity (Fig 4B) was a later event (observed at 8h), as it was in the EDL incubated with a high glucose medium (Fig 4A).

## Discussion

The biological relevance of AMPK activation, as well as how such regulation occurs, has been well described [13, 27–32]. In contrast, less is known about the factors that contribute to AMPK inhibition and its biological effects. We previously showed that incubation of rat EDL muscle for 1h in a high glucose (25mM) medium or a normoglycemic (5.5mM) medium to which a physiological concentration of leucine (100μM) had been added both decreased AMPK activity and impaired insulin signaling (Akt phosphorylation). In addition, we found that all of the changes, including increases in mTOR/p70S6K phosphorylation and protein synthesis, in addition to the insulin resistance, could be prevented by co-incubation with the AMPK activator AICAR. In the present study, we investigated the effects of these nutrients (25mM glucose or 100μM leucine) on AMPK activation and several factors that could modulate its activity, including serine phosphorylation at Ser^485/491^ and changes in SIRT1 and PP2A. We also sought to determine whether similar changes in these parameters occur in muscle *in vivo*, during a glucose infusion that increased plasma insulin as well as glucose.

We found that incubation of the EDL with 25mM glucose or 5.5mM glucose + leucine led to a 50% decrease in AMPK activity within 30min and an additional 50% reduction by 2h, as evidenced by decreases in both its phosphorylation at Thr^172^ and SAMS peptide phosphorylation. Interestingly, the later decreases in AMPK activity in these muscles were associated with increased phosphorylation of AMPK at Ser^485/491^ and changes in NAMPT and redox state at 1h, and alterations in SIRT1 expression (decreased) and PP2A activity (increased) at 2h. Similarly, *in vivo*, infusion of glucose at a rate that maintained plasma levels at 16–17mM and increased plasma insulin 30-fold diminished AMPK activity and its phosphorylation at Thr^172^ at 5h. In contrast to the findings in the incubated EDL, changes in Ser^485/491^ phosphorylation, SIRT1 abundance, NAMPT activity, and redox state were observed concurrently. Only PP2A activation was a later event (8h). These findings raise several fundamental questions: (1) by what mechanism do excesses of glucose and leucine lead to the initial decrease in AMPK activity in the EDL, (2) what is the physiological significance of the later decreases in AMPK activity and the changes in its putative regulators, and (3) why does the temporal sequence of events differ between incubated muscle and muscle from rats infused with glucose *in vivo* despite similar changes in all of the above-mentioned parameters?

To our surprise, in the incubated EDL, increases in most putative downregulators of AMPK activity occurred after, rather than before the initial decrease in AMPK activity (30min). Increased muscle glycogen content was the only regulatory factor we found to be changed at this early timepoint (Fig 3). Previous studies have shown that AMPK activation is lower in glycogen loaded versus depleted muscles in response to contraction [33] or AICAR [34] in rodents or in response to exercise in humans [35]. Furthermore, glycogen was recently shown to directly inhibit AMPK in a cell-free system by binding to the GBD of its β-subunit, thus causing an allosteric change that inhibits its phosphorylation at Thr^172^ [12]. Although further studies are required to determine whether glycogen is responsible for the initial inhibition of AMPK in our muscle incubation model, this data is consistent with the hypothesis of Mcbride et al. [12] that AMPK is sensitive not only to changes in immediately available cellular energy in the form of AMP and ATP (which were unchanged in our studies (Table 2)), but also to energy reserves in the form of glycogen. Despite the significant increase in muscle glycogen seen at 30min and 1h, no changes in phosphorylation of GSK3β were observed at any timepoint measured (30min-2h) (S1B Fig). This is somewhat surprising, since GSK3β contributes to the regulation of glycogen synthesis by phosphorylating and inhibiting glycogen synthase. However, transient changes in p-GSK3β may have preceded the increase in glycogen content and been missed in our studies.

Following these early changes, the next event observedwas an increase in AMPK Ser^485/491^ phosphorylation, which was increased by 250% after 1h of incubation in a high glucose medium (Fig 1C). Prior studies in other tissues suggest that phosphorylation at this site in response to other stimuli occurs very quickly (within 5–10min) and plays an important role in inhibiting AMPK [7, 9, 10, 36]. For example, Horman et al. [7] demonstrated in rat heart that at a normal glucose concentration, insulin increases αAMPK Ser^485/491^ phosphorylation within 5min, an effect mediated by Akt activation. Recently, we showed that insulin and IGF-1 also rapidly inhibit AMPK by such a mechanism in skeletal muscle and hepatocytes [9]. We observed a trend of increased Akt phosphorylation following 60min of incubation in high glucose (p = 0.12), but no changes were apparent at 30 or 120min (S1A Fig). This suggests that Akt may contribute to the increase in Ser^485/491^ in response to high glucose at 60min, although it does not rule out involvement of another kinase. In another series of investigations, Dagon et al [10] demonstrated that phosphorylation of α2 AMPK at Ser^491^ in response to leptin inhibits its activity in the hypothalamus. They also demonstrated that this increase in AMPK Ser^491^ phosphorylation is mediated by mTOR-p70S6 kinase and can be inhibited by the mTOR inhibitor rapamycin. Interestingly, in our study, incubation of the EDL with rapamycin had no effect on the ability of a high concentration of glucose to diminish AMPK phosphorylation at Thr^172^ or to stimulate phosphorylation at Ser^485/491^ (Fig 5). Potentially relevant to these observations are studies which have shown that the protein kinase C activator Phorbol-12-Myristate-13-Acetate (PMA) can diminish AMPK activity in cardiac or skeletal muscle cells [37, 38], and, where studied, it increased AMPK Ser^485/491^ phosphorylation [37]. Whether PKC activation contributed to AMPK Ser^485/491^ phosphorylation in the EDL muscle incubated with an elevated glucose concentration or in muscle of the glucose-infused rat has not yet been determined.

In the muscles incubated with high glucose or 5.5mM glucose + 100μM leucine, changes in NAMPT and redox state, as well as decreased SIRT1 expression (Figs 2 and 5), all of which also have been linked to diminished AMPK activity [19], occurred at various times after the decreases in Thr^172^ and SAMS peptide phosphorylation (both of which were observed at 30min). The reduction in SIRT1 abundance first became evident at 2h, well after the initial decrease in AMPK activity. Previous studies have revealed an interaction between SIRT1 and the AMPK kinase LKB1. For example, Lan et al.[15] demonstrated that overexpression of SIRT1 led to increased AMPK activity by causing translocation of LKB1 from the nucleus to the cytoplasm, where most of the cellular AMPK is located. Whether a decrease in SIRT1 leads to opposite changes and contributes to a reduction in AMPK activity is not known. Also unknown is why a normal physiological concentration of leucine affected all of these parameters in the same way as high glucose. We [6], and others [39, 40], have previously shown that leucine increases mTOR/P70S6K signaling, which can contribute to insulin resistance. However, as mentioned earlier, rapamycin did not affect the changes in AMPK phosphorylation in the present study. Whether these two nutrients bring about these changes through the same mechanisms requires further investigation.

Another finding in the incubated EDL and muscles of glucose infused rats was an increase in PP2A activity (Fig 4). The dephosphorylation of AMPK at Thr^172^ has been associated with the actions of various Ser/Thr protein phosphatases [22]. Wu and coworkers [11] found that a decrease in AMPK activity observed in bovine aortic endothelial cells after incubation with palmitate for 40h is associated with an increase in PP2A activation. Surprisingly, the increase in PP2A activity in the present study occurred much later (2h) than the initial decreases in AMPK Thr^172^ phosphorylation (30min). Hyperactivity of PP2A has been shown in muscle and other tissues in models of glucolipotoxicity and diabetes [41]. Since this is the last factor to change of those that were examined, one could speculate that the increase in PP2A activity produces a less reversible inhibition of AMPK by maintaining its dephosphorylation on Thr^172^, and possibly other sites.

*In vivo*, the infusion of glucose concurrently decreased AMPK activity (SAMS peptide assay and p-AMPK Thr^172^) and increased AMPK Ser^485/491^ phosphorylation in the red gastrocnemius (Fig 7). The co-occurrence of these changes *in vivo* differed from the delay in phosphorylation at Ser^485/491^ observed in the EDL incubated with high glucose. Although the precise explanation for this difference remains to be determined, one obvious factor could be the presence of insulin *in vivo*. As already noted, in heart and skeletal muscle, insulin has been shown both to increase AMPK Ser^485/491^ phosphorylation and decrease the phosphorylation of αAMPK at Thr^172^ and AMPK activity within 5–10min [7, 9]. Of further note, we observed changes in SIRT1 and related parameters at the same time as the changes in Thr^172^ and Ser^485/491^ phosphorylation in the muscle of glucose-infused rats. However, the increase in PP2A activity occurred much later, as it did in the EDL incubated in a high-glucose medium (without insulin). The presence of insulin and other circulating factors, as well as differences in animal age and weight could be responsible for the differences in the timing and sequence of changes between the two models; however, this remains to be determined.

In summary, we have demonstrated that incubation of EDL with either high glucose or leucine resulted in diminished AMPK Thr^172^ phosphorylation and activity that continued to decrease over time. An *in vivo* glucose infusion also caused a reduction in AMPK activation along with changes in other putative regulators of the enzyme. In all three models, concurrent or sequential changes in αAMPK Ser^485/491^ phosphorylation (increased), SIRT1 protein level (decreased), and PP2A activity (increased) were observed. Though all of these factors may diminish AMPK activity, glycogen content was the only regulatory factor found to be changed at 30 min in the incubated EDL. These data suggest that the initial inhibition of AMPK in response to high glucose or leucine may be due to sensing an increase in cellular energy state in the form of glycogen, though further studies are required to confirm this hypothesis. The physiological and/or pathophysiological significance of the later occurring changes that correlate with additional decreases in AMPK activity also requires further investigation. An interesting possibility is that they contribute to more long-lasting changes that could alter gene expression or mitochondrial function and are less readily reversible. Future studies with an expanded time course in which these factors are individually inhibited or knocked down could address these questions.

## Supporting Information

S1 Fig Effects of incubation with 25mM glucose on Akt and GSK3β phosphorylation. EDL muscles incubated for 60 min in media containing 5.5 or 25 mM glucose for 30, 60 or 120 min. Muscle lysates were analyzed for P-Akt Ser^473^ (A) or P-GSK3β Ser^9^ (B) by western blot. Results show quantification of western blots by densitometry. Results are means ± SE (n = 4).
(TIF)
