# Supplementary figures and images for: Nutrient Excess and AMPK Downregulation in Incubated Skeletal Muscle and Muscle of Glucose Infused Rats
Source: PLoS One. 2015 May 21;10(5):e0127388. doi: 10.1371/journal.pone.0127388 (PMC4440828; doi:10.1371/journal.pone.0127388)

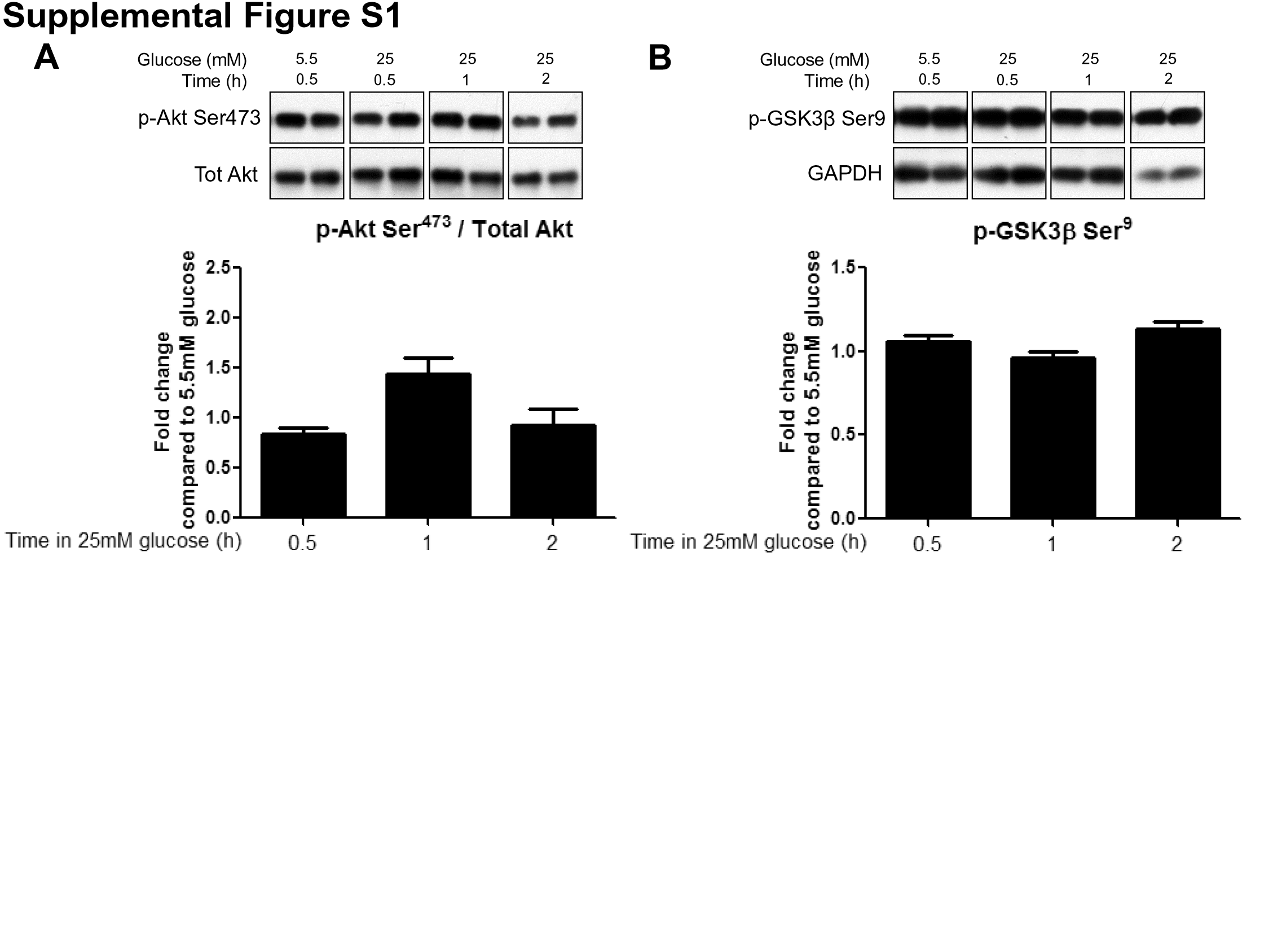

Supplement: S1 Fig — EDL muscles incubated for 60 min in media containing 5.5 or 25 mM glucose for 30, 60 or 120 min. Muscle lysates were analyzed for P-Akt Ser473 (A) or P-GSK3β Ser9 (B) by western blot. Results show quantification of western blots by densitometry. Results are means ± SE (n = 4). (TIF) [file pone.0127388.s001.tif]
